# Supplementary material for: Inferring characteristics of bacterial swimming in biofilm matrix from time-lapse confocal laser scanning microscopy
Source: eLife. 2022 Jun 14;11:e76513. doi: 10.7554/eLife.76513 (PMC9273218; doi:10.7554/eLife.76513)
Supplement: Transparent reporting form [file elife-76513-transrepform1.pdf]

eLIFE
1st Floor
24 Hills Road
Cambridge CB2 1JP, UK
P 01223 855340
W elifesciences.org
T @elife

eLife's transparent reporting form

We encourage authors to provide detailed information *within their submission* to facilitate the interpretation and replication of experiments. Authors can upload supporting documentation to indicate the use of appropriate reporting guidelines for health-related research (see EQUATOR Network), life science research (see the BioSharing Information Resource), or the ARRIVE guidelines for reporting work involving animal research. Where applicable, authors should refer to any relevant reporting standards documents in this form.

If you have any questions, please consult our Journal Policies and/or contact us: editorial@elifesciences.org.

Sample-size estimation

- You should state whether an appropriate sample size was computed when the study was being designed
- You should state the statistical method of sample size computation and any required assumptions
- If no explicit power analysis was used, you should describe how you decided what sample (replicate) size (number) to use

Please outline where this information can be found within the submission (e.g., sections or figure legends), or explain why this information doesn't apply to your submission:

In the Bayesian inference, the sample number is the total number of time points among the whole set of swimmer trajectories, which can not be controlled experimentally. Hence, no explicit power analysis was conducted previously to the experimental design. Three biological replicates have been performed for image acquisition in accordance with usual practices in the field. The acquisition focal plan has been selected near the well boundary, where the well wall physically constrains the swimmer trajectories in one direction, which select longer trajectories in the 2D plane, increasing the number of observed time points. The resulting number of time points by batch can be found in Table 1.

Replicates

- You should report how often each experiment was performed
- You should include a definition of biological versus technical replication
- The data obtained should be provided and sufficient information should be provided to indicate the number of independent biological and/or technical replicates
- If you encountered any outliers, you should describe how these were handled
- Criteria for exclusion/inclusion of data should be clearly stated
- High-throughput sequence data should be uploaded before submission, with a private link for reviewers provided (these are available from both GEO and ArrayExpress)

Please outline where this information can be found within the submission (e.g., sections or figure legends), or explain why this information doesn't apply to your submission:

eLife Sciences Publications, Ltd is a limited liability non-profit non-stock corporation incorporated in the State of Delaware, USA, with company number 5030732, and is registered in the UK with company number FC030576 and branch number BR015634 at the address 1st Floor, 24 Hills Road, Cambridge CB2 1JP | August 2014

Three biological replicates (i.e. microbiological culture, biofilm growth, swimmer labeling, swimmer infiltration of biofilms and image acquisition) have been performed for each swimmer species. No technical replication was needed.
Trajectories with less than 8 time points were considered too short after acceleration computation and filtered out.
These informations are available in the Material and Method, subsection 'Infiltration of host biofilms by bacilli swimmers', 'Confocal Laser Scanning Microscopy (CLSM)' and 'Post-processing of image data'.
Post-processed image data (i.e. swimmer position, speed, acceleration, biofilm density and biofilm density gradient at swimmer position) are available at https://forgemia.inra.fr/bioswimmers/swim-infer/SwimmerData, alongside with a jupyter notebook providing a tutorial for data visualization.

eLife Sciences Publications, Ltd is a limited liability non-profit non-stock corporation incorporated in the State of Delaware, USA, with company number 5030732, and is registered in the UK with company number FC030576 and branch number BR015634 at the address 1st Floor, 24 Hills Road, Cambridge CB2 1JP | August 2014

Statistical reporting

- Statistical analysis methods should be described and justified
- Raw data should be presented in figures whenever informative to do so (typically when N per group is less than 10)
- For each experiment, you should identify the statistical tests used, exact values of N, definitions of center, methods of multiple test correction, and dispersion and precision measures (e.g., mean, median, SD, SEM, confidence intervals; and, for the major substantive results, a measure of effect size (e.g., Pearson's r, Cohen's d)
- Report exact p-values wherever possible alongside the summary statistics and 95% confidence intervals. These should be reported for all key questions and not only when the p-value is less than 0.05.

Please outline where this information can be found within the submission (e.g., sections or figure legends), or explain why this information doesn't apply to your submission:

The inference method is precisely described in the Material and Methods, subsection 'Inference'. Parameter estimation reports provide mean, standard deviation and confidence intervals.
Additional statistics are described in the subsection 'Plot and statistics'. Pairwise comparisons between 1D distributions means are performed using T-tests and the resulting p-values are gathered in Annex 1, table 1.

(For large datasets, or papers with a very large number of statistical tests, you may upload a single table file with tests, Ns, etc., with reference to sections in the manuscript.)

Group allocation

- Indicate how samples were allocated into experimental groups (in the case of clinical studies, please specify allocation to treatment method); if randomization was used, please also state if restricted randomization was applied
- Indicate if masking was used during group allocation, data collection and/or data analysis

Please outline where this information can be found within the submission (e.g., sections or figure legends), or explain why this information doesn't apply to your submission:

Groups are defined accordingly to the bacterial swimmer species.

Additional data files ("source data")

- We encourage you to upload relevant additional data files, such as numerical data that are represented as a graph in a figure, or as a summary table
- Where provided, these should be in the most useful format, and they can be uploaded as "Source data" files linked to a main figure or table
- Include model definition files including the full list of parameters used
- Include code used for data analysis (e.g., R, MatLab)
- Avoid stating that data files are "available upon request"

Please indicate the figures or tables for which source data files have been provided:

eLIFE
1st Floor
24 Hills Road
Cambridge CB2 1JP, UK
P 01223 855340
W elifesciences.org
T @elife

Image data needed for the computation of Figure 2 are available at https://forgemia.inra.fr/bioswimmers/swim-infer/SwimmerData, alongside with a jupyter notebook providing a tutorial to build Figure 2.
Inference and model codes are provided at https://forgemia.inra.fr/bioswimmers/swim-infer/. A second jupyter notebook allows the production of synthetic data, inference and comparison of the fitted model with the original synthetic data.

eLife Sciences Publications, Ltd is a limited liability non-profit non-stock corporation incorporated in the State of Delaware, USA, with company number 5030732, and is registered in the UK with company number FC030576 and branch number BR015634 at the address 1st Floor, 24 Hills Road, Cambridge CB2 1JP | August 2014
